# Cervical Gene Delivery of the Antimicrobial Peptide, Human β-Defensin (HBD)-3, in a Mouse Model of Ascending Infection-Related Preterm Birth

**DOI:** 10.3389/fimmu.2020.00106

**Published:** 2020-02-11

**Authors:** Natalie Suff, Rajvinder Karda, Juan Antinao Diaz, Joanne Ng, Julien Baruteau, Dany Perocheau, Peter W. Taylor, Dagmar Alber, Suzanne M. K. Buckley, Mona Bajaj-Elliott, Simon N. Waddington, Donald Peebles

**Affiliations:** ^1^Gene Transfer Technology Group, Department of Maternal and Fetal Medicine, Institute for Women's Health, University College London, London, United Kingdom; ^2^Preterm Birth Group, Department of Maternal and Fetal Medicine, Institute for Women's Health, University College London, London, United Kingdom; ^3^Preterm Birth Group, Department of Women and Children's Health, King's College London, St Thomas' Hospital, London, United Kingdom; ^4^Metabolic Medicine, Great Ormond Street Hospital for Children NHS Foundation Trust, London, United Kingdom; ^5^University College London School of Pharmacy, London, United Kingdom; ^6^Great Ormond Street Institute of Child Health, London, United Kingdom; ^7^SA/MRC Antiviral Gene Therapy Research Unit, Faculty of Health Sciences, University of the Witwatersrand, Johannesburg, South Africa

**Keywords:** cervix, gene therapy, preterm birth, antimicrobial peptides, ascending infection

## Abstract

Approximately 40% of preterm births are preceded by microbial invasion of the intrauterine space; ascent from the vagina being the most common pathway. Within the cervical canal, antimicrobial peptides and proteins (AMPs) are important components of the cervical barrier which help to prevent ascending vaginal infection. We investigated whether expression of the AMP, human β-defensin-3 (HBD3), in the cervical mucosa of pregnant mice could prevent bacterial ascent from the vagina into the uterine cavity. An adeno-associated virus vector containing both the *HBD3* gene and *GFP* transgene (AAV8 HBD3.GFP) or control AAV8 GFP, was administered intravaginally into E13.5 pregnant mice. Ascending infection was induced at E16.5 using bioluminescent *Escherichia coli* (*E. coli* K1 A192PP-lux2). Bioluminescence imaging showed bacterial ascent into the uterine cavity, inflammatory events that led to premature delivery and a reduction in pups born alive, compared with uninfected controls. Interestingly, a significant reduction in uterine bioluminescence in the AAV8 HBD3.GFP-treated mice was observed 24 h post-*E. coli* infection, compared to AAV8 GFP treated mice, signifying reduced bacterial ascent in AAV8 HBD3.GFP-treated mice. Furthermore, there was a significant increase in the number of living pups in AAV HBD3.GFP-treated mice. We propose that HBD3 may be a potential candidate for augmenting cervical innate immunity to prevent ascending infection-related preterm birth and its associated neonatal consequences.

## Introduction

Preterm birth, defined as delivery before 37 completed weeks gestation, affects 11% of pregnancies worldwide (1). It is the single largest cause of mortality in infants under 5 years old and it is associated with serious morbidity in the surviving infants, particularly for those born before 32 weeks gestation (2). Prematurity accounts for 29% of global neonatal deaths per year and for 3.1% of total disability adjusted life years in the global burden of disease (3). Despite extensive research, the rates of preterm birth have remained stable over the years; this is thought to be largely due to a lack of effective preventative treatments.

Preterm birth is a highly complex, multifactorial biological process which culminates in the premature activation of the common parturition pathway (4). Evidence indicates a role for infection and inflammation in preterm birth, particularly in those occurring before 28 weeks, and it is estimated to be associated with up to 40% of preterm deliveries (5). In clinical studies, the increased prevalence and diversity of intrauterine bacterial DNA is associated with preterm pre-labor rupture of membranes and spontaneous preterm birth (6). Bacteria linked with preterm birth include those genera associated with relatively low pathogenicity such as *Ureaplasma, Fusibacterium, Mycoplasma* and *Streptococcus* (6). Once in the pregnant uterus, bacteria interact with the mucosal lining and the local immune system initiating an inflammatory cascade leading to cervical ripening and myometrial contractility, ultimately resulting in preterm parturition (7). Animal models confirm this link; inoculation of the intrauterine cavity with live bacteria or bacterial pattern recognition pattern (PRR) moieties, such as lipopolysaccharide (LPS), leads to preterm birth (8, 9). In humans, this preterm inflammatory pathway has proved resistant to most therapies including antibiotics, cervical cerclage and tocolytics; meta-data analysis shows that progesterone appears to delay preterm birth, although its role in infection and inflammation remains unclear (10).

Ascending vaginal infection is considered to be the most common route by which bacteria gain access into the uterine cavity in cases of spontaneous preterm birth (11). This hypothesis is supported by the association recorded between the bacterial species identified in the amniotic fluid, fetal membranes and placenta and those normally found in the lower genital tract (6, 12). Additionally, recent data suggests that dominance of a particular bacterial species within the vaginal microbiota are associated with an increased risk of preterm birth (13).

The ability of the human cervix to prevent bacteria ascending from the vagina to the uterine cavity depends on several factors, including a mucus plug that provides a negatively-charged platform for interaction with cationic antimicrobial peptides (AMPs) including the constitutively expressed human β-defensins-1 (14, 15). It is not known why certain women develop ascending infection; women who have had previous cervical excisional treatment are associated with an increased risk of preterm birth (16), but it is likely that an endogenous compromise in cervical mucosal immunity plays a key role in these cases (17). In support of this, recent evidence has shown an association between PTB and low mid-trimester cervico-vaginal levels of human β-defensin 2 (18).

HBDs, produced by the cervical epithelium, critical for maintaining mucosal host-microbial homeostasis (19). In addition to their direct microbicidal role against pathogens, they mediate inflammation by influencing cytokine production, immune cell chemotaxis and epithelial cell proliferation (20). Human β-defensin 3 (HBD3) has potent and broad-spectrum antimicrobial activity against bacteria, fungi, and viruses (21, 22). Possessing multiple positively charged arginine and lysine residues it has the highest net positive charge of all AMPs which probably contributes to its broad-spectrum action. Its antimicrobial activity is not affected by differing physiological salt concentrations, making it an ideal choice for a clinical treatment (21). Furthermore, HBD3 binds to bacterial products, such as LPS, resulting in reduced pro-inflammatory cytokine responses (23).

The transfer and expression of therapeutic genes in the context of cervical mucosa has been explored as a potential treatment for infectious diseases. For example, anti-HIV antibodies have been delivered using an adeno-associated viral vector (AAV) to human cervical and vaginal cells *in vitro* and also to the lower genital tract of rhesus macaques to successfully prevent mucosal acquisition of HIV infection *in vivo* (24, 25). Gene transfer to the murine cervix, however, has not been described before, although the use of adenoviral vectors to deliver specific pro-inflammatory cytokines to the mouse vagina has been explored as a possible treatment for vaginal candidiasis (26).

The objectives of our present study were to evaluate the function of the HBD3 gene, delivered to the cervix using AAV8, in a mouse model of ascending infection-related preterm birth. We have previously established an ascending infection-model of preterm birth using bioluminescent *E. coli* K1 A192PP-*lux2* (*E. coli* K1) (27, 28), a strain known to cause neonatal sepsis and meningitis in rats. In this model, *E. coli K1* administration induced delivery significantly earlier than in pregnant mice receiving intravaginal PBS as well as leading to a significant reduction in the proportion of pups born alive compared with intravaginal PBS controls (28).

Here, we tested the hypothesis that cervical gene expression of HBD3 reduces microbial ascension into the pregnant uterine cavity, reducing the frequency of preterm birth.

## Materials and Methods

### Viral Vector Production

The following viral vectors were used in this study; AAV2/8, VSV-g pseudotyped lentivirus, recombinant adenovirus serotype 5. The vectors carried the firefly luciferase or enhanced GFP transgene under the control of the cytomegalovirus (CMV) promoter. Lentiviral vectors were gifted from Dr. Stephen Howe (University College London, UK). The adenoviral vectors were gifted from Dr. Alan Parker (Cardiff University, UK). The AAV viral vectors were developed and purchased from Vector Biolabs (Malvern, USA).

An AAV8 bicistronic vector encapsidating a single-stranded DNA sequence containing the HBD3 gene (Vega Sanger HBD3 transcript VEGA68:CM000670.2) under the transcription activity of the CMV promoter, followed by the eGFP gene under the transcription activity of a further CMV promoter, and BGH polyA downstream from the HBD3 and eGFP gene was generated (Vector Biolabs, Malvern, USA) (Supplementary Figure 1).

### Animals and Treatments

All animal studies were conducted under UK Home Office license 70/8030 and approved by the UCL Ethical Review Committee.

C57BL/6N-Tyr^c−Brd^ mice were obtained from the Charles River Laboratory (Oxford, UK) and adult mice (6–12 weeks) were time mated. The following morning (when a vaginal plug was noted) was designated as embryonic day 0.5.

#### Animal Injections

Adult (6–8 weeks old) female C57BL/6N-Tyr^c−Brd^ mice were anesthetized with isoflurane in 100% oxygen. Ten microliter of virus (diluted in PBS if necessary) to a concentration of 1 × 10^12^ genomic copies/mL was administered intravaginally using a 200 μl sterile pipette tip. This was applied in combination with 20 μl of AK12 thermosensitive pluronic gel (PolySciTech, Indiana, U.S.A)

For pregnancy experiments, vector was administered as described above on embryonic day 13.5.

#### Ascending Vaginal Infection Model

The ascending vaginal infection model was developed using *E. coli* K1 A192PP modified to contain the *lux* operon from *Photorhabdus luminescens* (*E. coli* K1 A192PP-*lux2*). Twenty microliter of mid-logarithmic-phase *E. coli* (1 × 10^2^ *E. coli* K1 A299 resuspended in 10 mM phosphate buffer), or 20 μl of phosphate-buffered saline (PBS) in control animals, was delivered into the vagina of pregnant mice anesthetized with isoflurane using a 200-ml pipette tip on embryonic day 16.5.

Following bacterial administration, mice were placed in individual cages and continuously monitored with individual closed-circuit television cameras and a digital video recorder (29). Time to delivery was recorded and defined as the number of hours from the time of bacterial administration to delivery of the first pup. The number of live and dead pups were recorded. Living pups were weighed daily and were culled if there was a 10% loss in body weight (in accordance with PPL 70/8030).

#### Whole-Body Bioluminescence Imaging

Adult mice were anesthetized with isoflurane in 100% oxygen. Neonatal mice (up to postnatal day 6) remained conscious during imaging (30). Mice were imaged using a cooled charged-coupled device camera (IVIS machine, Perkin Elmer, Coventry, UK) for between 1 s and 5 min. The regions of interest (ROI) were measured using Living Image Software (Perkin Elmer) and expressed as photons per second per centimeter squared per steradian (photons/second/cm^2^/sr).

### Tissue Collection

Non-pregnant mice were sacrificed 72 h after viral vector administration. Pregnant mice were sacrificed 18 and 24 h after intravaginal infection. Mice were anesthetized using isoflurane, the right atrium incised, and PBS injected into the left ventricle for exsanguination. Uterine tissue was stored in 4% paraformaldehyde. Embryos were stored in 10% neutral-buffered formalin. A separate cohort of mice were sacrificed by cervical dislocation and vagina, cervix, uterus, placenta, and fetal membranes were collected and stored at −20°C for protein analysis.

### Storage of Fixed Cervical and Vaginal Tissues

Cervical and vaginal tissues were stored in 4% PFA for 48 h, transferred to 30% sucrose at 4°C then 40 μm transverse sections obtained using a microtome. Embryos were stored in 10% neutral-buffered formalin for 48 h, followed by storage in 70% ethanol before paraffin embedding and sectioning at 5 μm.

### *Ex vivo* Luminometry

Tissue samples were lysed with 500 μl of 1x Lysis buffer (Promega) followed by homogenisation. The homogenates were centrifuged for 10 min at 18,000 g and the supernatants collected. Each sample was loaded on to a white 96 well plate. 1.5 mM of luciferase (Promega) was added at a 1:1 volume ratio to the sample. A FluoStar Omega microplate reader (BMG labtech) was used to read the luminescence and the results were analyzed using MARS data analysis data software (BMG labtech).

### HBD3 Enzyme-Linked Immunosorbent Assay (ELISA)

HBD3 concentrations in vaginal lavage was measured by ELISA (PeproTech, London, UK) per manufacturer's instructions. Results were read using the FluoStar Omega microplate reader and analyzed in MARS data analysis data software. The cross-reactive non-specific background from the ELISA was not subtracted from our analysis so that others repeating the ELISA have some measure of the degree of background.

### GFP Immunohistochemistry and Co-localization Immunofluorescence

Representative sections of the organ were selected, mounted onto double-coated chrome gelatin Superfrost slides (VWR, Leicestershire, UK) and left to dry. The slides were placed in 4% PFA for 10 min followed by washing in TBS (1x Tris-buffered saline). They were treated with 30% H_2_O_2_ in TBS for 30 min at room temperatures and then blocked with 15% normal goat serum (Vector Laboratories, Peterborough, U.K.) in 0.1% TBS-T (0.1% of Triton X-100 in 1x TBS) for 30 min at room temperature. This was followed by incubation in primary anti-GFP antibody (Abcam, Cambridge, U.K.) in 10% normal goat serum in 0.1% TBS-T overnight at 4°C. The slides were washed and secondary antibody (Abcam, Cambridge, U.K.) in 10% serum in 0.1% TBS-T was added for 2 h at room temperature. Following this the slides were incubated for 2 h in ABC Vectastain (Vector Labs, Peterborough, UK). Slides were transferred into DAB solution (0.05% 3.3′-diaminobenzidine (DAB) in TBS with 30% H_2_O_2_ and left for 2 to 3 min. The slides were air dried, dehydrated in 100% ethanol and placed in Histoclear (National diagnostics, USA), followed by cover slipping with DPX mounting solution.

A similar protocol was followed for co-localization immunofluorescence staining; slides were incubated in two primary antibodies (in 10% normal goat serum in 0.1% TBS-T); anti-GFP antibody (Abcam, Cambridge UK) and anti-pan cytokeratin antibody (Abcam, Cambridge, UK) or anti-HBD3 antibody (Abcam, Cambridge, UK). This was followed by incubation with two corresponding Alexafluor secondary antibodies (Abcam, Cambridge, U.K.) in 10% normal goat serum in 0.1% TBS-T. Sections were then incubated with DAPI for 2–3 min and then washed in TBS. The sections were dried away from direct sunlight and then coverslips mounted using Fluoromount-G. Sections were stored at 4°C.

### Neutrophil and Monocyte Immunohistochemistry and Influx Quantification

Neutrophil immunohistochemistry was performed using the same protocol to GFP immunohistochemistry above; with primary rat anti-Ly6g antibody and Goat anti-rat secondary antibody (Abcam, Cambridge, U.K.).

Cervical sections were visualized using a x5 objective lens and the epithelium and sub-epithelial stromal areas were identified. Five random fields of view were selected using a x40 objective lens. The numbers of neutrophils were counted per area. Three to five sections were counted per mouse and the number of neutrophils counted per area averaged per mouse.

### *E. coli* Immunofluorescence

Formalin-fixed embryos were paraffin embedded and sectioned. Paraffin embedded slides were placed in Histoclear for 10 min and then rehydrated in ethanol. Antigen retrieval was then performed by boiling the slides in citrate buffer for 20 min followed by washing in 1xTBS. Slides were blocked with 15% normal goat serum in 0.1% TBS-T for 30 min at room temperature. This was followed by incubation in primary rabbit anti-*E. coli* antibody (Abcam, Cambridge, U.K.) in 10% normal goat serum in 0.1% TBS-T overnight at 4°C. The slides were washed and secondary Goat anti-rabbit IgG H&L Alexa Fluor® 488 (Abcam, Cambridge, U.K.) antibody in 10% serum/ 0.1% TBS-T was added for 2 h at room temperature. Following this the slides were incubated for 2 h in ABC Vectastain (Vector Labs, Peterborough, UK). Slides were transferred into DAB solution (0.05% DAB/30% H_2_O_2_/TBS) and left for 2–3 min. The slides were air dried, dehydrated in 100% ethanol and placed in Histoclear (National diagnostics, USA), followed by cover slipping with DPX mounting solution.

### *E. coli* Killing Assays

*Escherichia coli* K1 was grown to mid-logarithmic phase and diluted to 1 × 10^5^ colony forming units(CFU)/ml. The bacteria were centrifuged at 14,000 g for 3 min. The pellet was washed once in 10 mM phosphate buffer followed by further centrifugation and re-suspension in 10 mM phosphate buffer. In a 96 well plate, 90 μl of the resuspended bacteria was mixed with 90 μl vaginal lavage or 10 mM phosphate buffer. The plate was incubated for 30 mi at 37°C. Twenty microlitre of each sample was then mixed with PBS (to inhibit further AMP activity). Serial dilutions were then plated and placed at 37°C overnight. CFUs were counted the following morning.

### Cervical Inflammatory Cytokine Analyses by Quantitative PCR

A cohort of cervices initially collected at 72 h post viral vector administration (as detailed above) were stored in *RNAlater* at −80°C for quantitative PCR (qPCR) analysis. Total RNA was extracted using the RNeasy mini kit (Qiagen, UK), as per the manufacturer's guidelines. Total RNA was reverse transcribed with the High Capacity cDNA Reverse Transcription kit (Applied Biosystems, USA). Primer sets were obtained from Life Technologies (Supplementary Table 1) and qPCR was performed in the presence of SYBR green. Target gene expression was normalized for RNA loading by using *GAPDH*, using the 2^−ΔΔCt^ method of analysis. All qPCR analyses were performed on an Applied Biosystems QuantStudio 3 instrument (Applied Biosystems, USA).

### Bacterial DNA Extraction

Bacterial DNA extraction from frozen vaginal lavage samples was done using Qiagen spin protocol as per manufacturer's instructions with an additional bead beating step (Qiagen DNA mini kit, Denmark).

### 16S DNA Sequencing

The DNA from the above step was quantified using a Qubit DNA high sensitivity assay kit and Qubit 2.0 machine (Thermo Fisher Scientific, UK). The DNA concentration in each well was normalized to the lowest concentration sample. The DNA was then pooled including negative DNA extraction controls. This library was diluted to 0.4 nM after quantification using the Qubit 2.0, standard curve qPCR and an Agilent high sensitivity DNA kit with the Agilent 2200 Tapestation instrument (Agilent genomics, Santa Clara, US). Library preparation was carried out using dual-indexed forward and reverse primers, with barcodes. Library preparation PCR was performed. The resulting amplicon was cleaned and pooled using AMPure XP beads (Beckman Coulter) as per manufacturer's instructions. Each plate was pooled into an equimolar final library after quantification using a Qubit 2.0 (Life technologies). Library was loaded onto a MiSeq (Illumina) as per manufacturer's protocol for 500 cycle V2 kits with the addition of custom sequencing primers for read 1, read 2 and index 1. Data was analyzed using QIIME software (v1.8.0).

### Statistics

Data are expressed as means ± SEM. Time-to-delivery data were log-transformed before analysis, and the proportion of live born pups was arc-sin transformed before analysis. Data were analyzed by unpaired *t*-tests, one-way ANOVAs and two-way ANOVAs (with *post-hoc* Bonferroni tests). All statistical analyses were performed with GraphPad Prism software version 7.0. *P* < 0.05 was considered statistically significant.

## Results

### A Murine Model of Ascending Bacterial Infection and Preterm Birth

**Figure 1:**
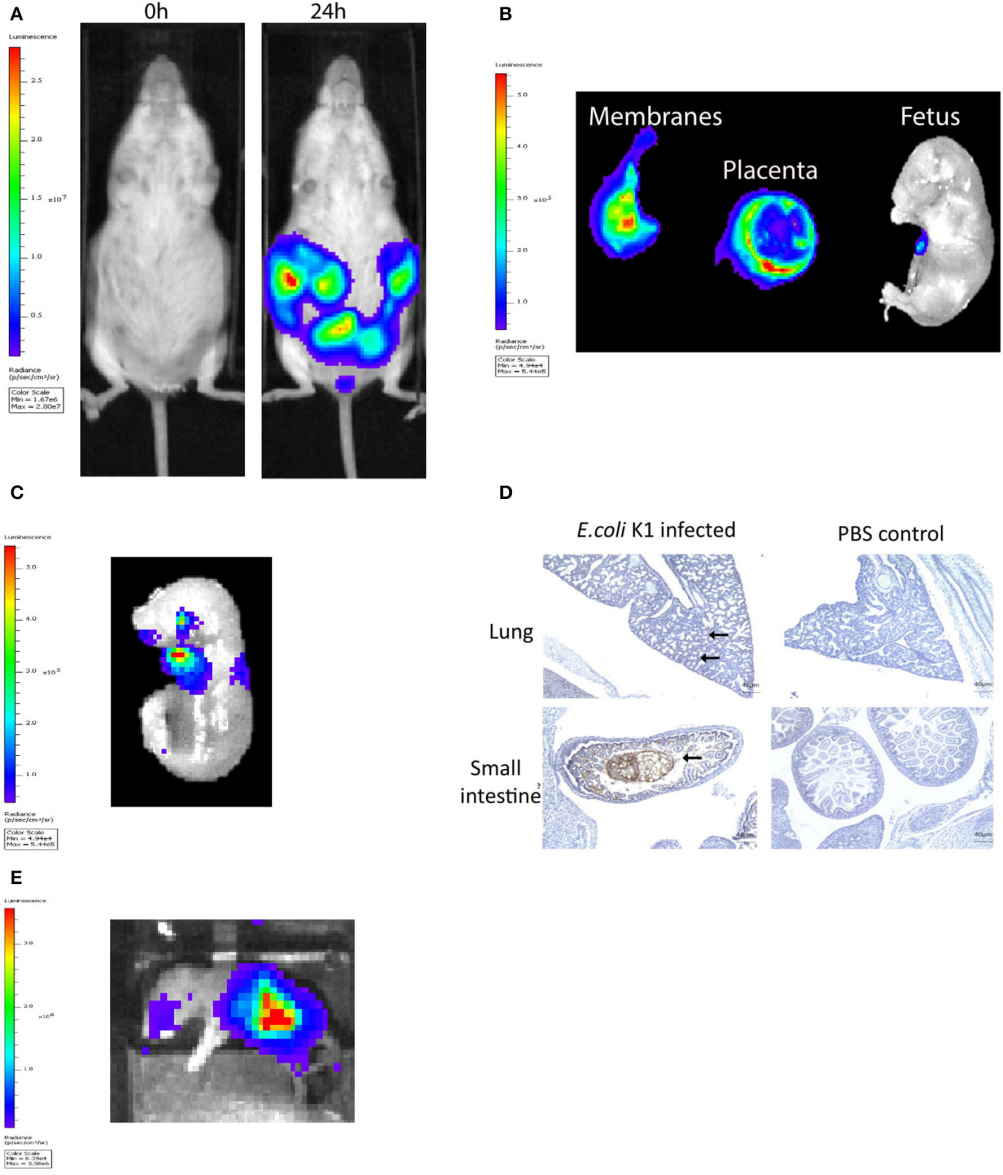
Intravaginal bioluminescent *Escherichia coli* K1 A192PP-lux2 (*E. coli* K1) can ascend into the pregnant uterine cavity and induce premature delivery (30). **(A)** Pregnant mice received intravaginal *E. coli* K1 on embryonic day 16.5, bacteria ascend into the uterine cavity over 24 h. **(B)** At 18 h after *E. coli* K1 administration bacteria is seen specifically within the pregnant uterine cavity and is detected in the fetal membranes, the placenta and the amniotic fluid by 18 h. **(C)** By 24 h, bacteria is detected in the fetus. **(D)** Immunohistochemical detection of *E. coli* in the fetus shows *E. coli* specifically within the lung alveoli and small intestine at 24 h, whilst no *E. coli* is seen in the uninfected fetus (sections counterstained with haematoxylin), *n* = 3, Scale bar 40 μm. **(E)** By Postnatal day 1 (72 h after infection), *E. coli* is clearly seen in the Gastrointestinal tract.

The murine model of infection was essentially as described previously by our laboratory (28). The ability of a pathogenic strain of *E. coli* K1 A192PP-*lux2* (*E. coli* K1) to ascend into the embryonic day 16.5 pregnant uterine cavity was investigated (28). Bioluminescence imaging of the dam revealed ascent of bacteria to the top of the uterine cavity by 24 h (Figure 1A), with diffuse spread of bacteria in the fetal membranes, the placenta and the amniotic fluid by 18 h (Figure 1B). By 24 h bacteria were evident in the fetus (Figure 1C); immunoperoxidase staining for bacteria revealed microbial presence within the respiratory and gastro-intestinal (GI) tract (Figure 1D). By postnatal day 1 (~72 h post-infection), bacteria were predominantly seen in the GI tract (Figure 1E).

### Selection of Viral Vector for Optimal Gene Transfer to the Cervical Mucosa

**Figure 2:**
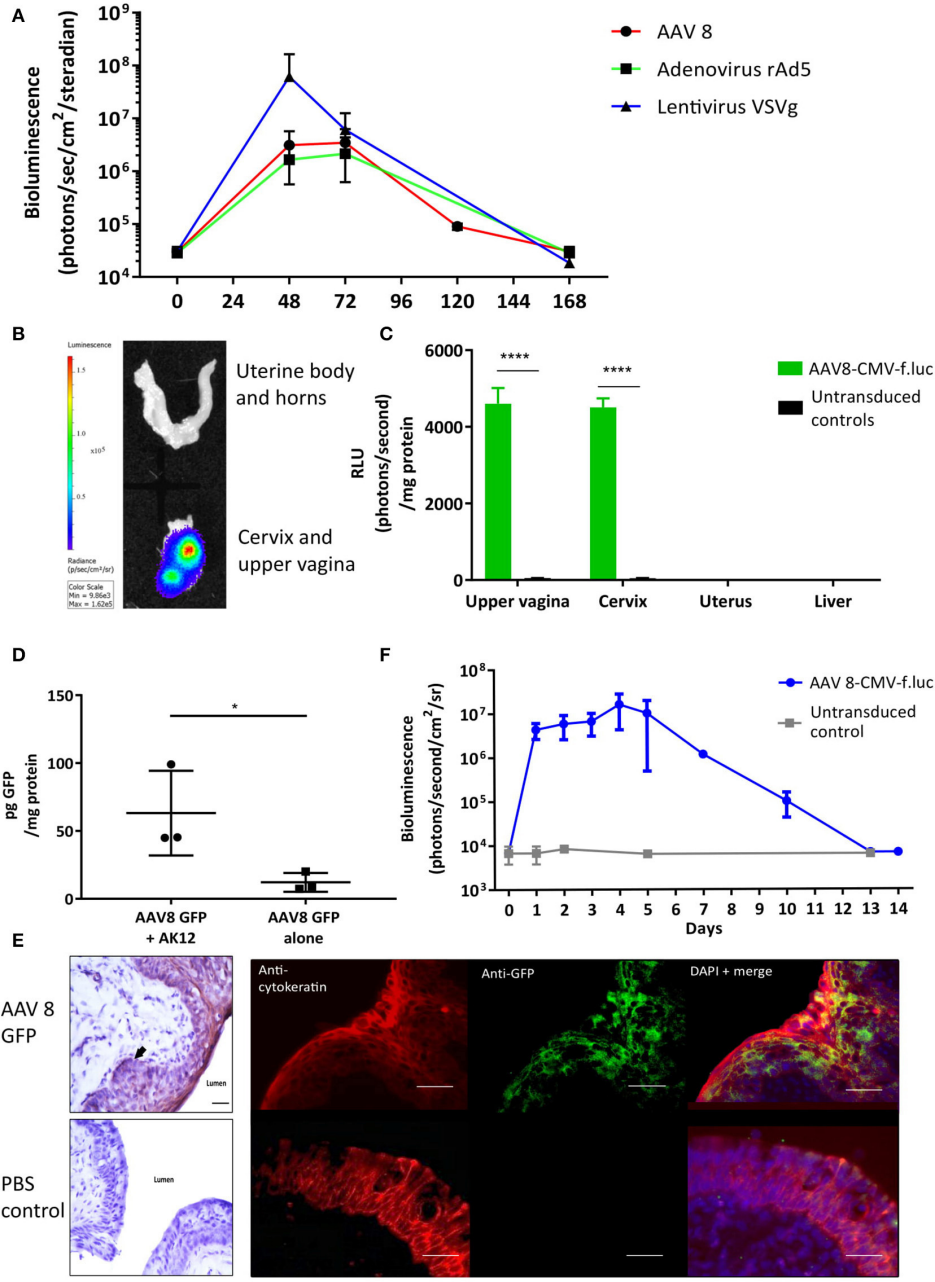
Gene delivery to the cervix is possible using viral vectors. **(A)** Adenovirus-associated virus-8 (AAV-8), Recombinant adenovirus 5 (rAd5), VSVg lentiviral vectors can deliver luciferase to the cervix resulting in transient luciferase expression. **(B,C)** 72 h following AAV8-CMV-f.luc administration, luciferase expression is limited to the cervix and upper vagina, *n* = 3. ^****^P < 0.0001, data were analyzed by a 1-way ANOVA with *post-hoc* Bonferroni test. Gene transfer to the cervix using AAV 8 viral vector is improved when delivered with the pluronic gel, AK12, and results in transient protein expression in the epithelial cell layers. **(D)** Cervical GFP expression is increased when AAV8 GFP is delivered in combination with AK12, compared with AAV8 GFP delivery alone, *n* = 3. ^*^P < 0.05, data were analyzed by a unpaired *T*-test. **(E)** GFP expression is detected in the epithelial layers of the cervix, confirmed by protein co-localization with cytokeratin expression. Scale bar 20 μm. **(F)** Following delivery of AAV8-CMV-f.luc in combination with AK12 gel, luciferase expression in the cervix lasted for up to 14 days, with peak expression occurring between 3 and day 5, *n* = 3.

To determine if viral vectors are capable of delivering genes to the cervical mucosa, separate cohorts of non-pregnant adult mice received intravaginal delivery of either adeno-associated virus serotype 2/8 (AAV8), recombinant adenovirus serotype 5 (rAD5) and VSVg pseudotyped HIV lentivirus, each containing the firefly luciferase transgene. Luciferase expression was seen in the lower genital tract 48 to 120 h after vector administration (Figure 2A). Although there was high luciferase activity from VsVG pseudo-typed HIV lentivirus, AAV was chosen for the ensuing studies due to its relatively low immunogenicity, episomal-nature, thermostability and success in numerous clinical trials (31–33). Following AAV8 administration, it was confined to the upper vagina and cervix at 72 h with no spread to the uterus or liver (Figures 2B,C). This vector was used for the remaining experiments due to its relatively low immunogenicity and success in a wide range of pre-clinical and clinical trials (31, 34).

### Specific Location of Vaginal and Cervical Protein Expression

Thermosensitive pluronic gels have been developed for numerous functions, including vaginal drug delivery (35–37). We assessed AK12, that gels at 30 degrees Celsius, as a method to prolong the contact time of the vector with the epithelium to improve transduction. Delivering AAV8 GFP in combination with this gel intravaginally resulted in significantly higher GFP expression than delivering vector alone (*P* = *0.02*, Figure 2D). This expression was co-localized with cytokeratin, a marker of the cervical epithelial cell layers (Figure 2E). Following AAV-8 luciferase intravaginal administration bioluminescence imaging revealed luciferase expression to behighest between 3 and 5 days after transduction and lastingfor up to 14 days (Figure 2F).

### HBD3 Expression and Function

**Figure 3:**
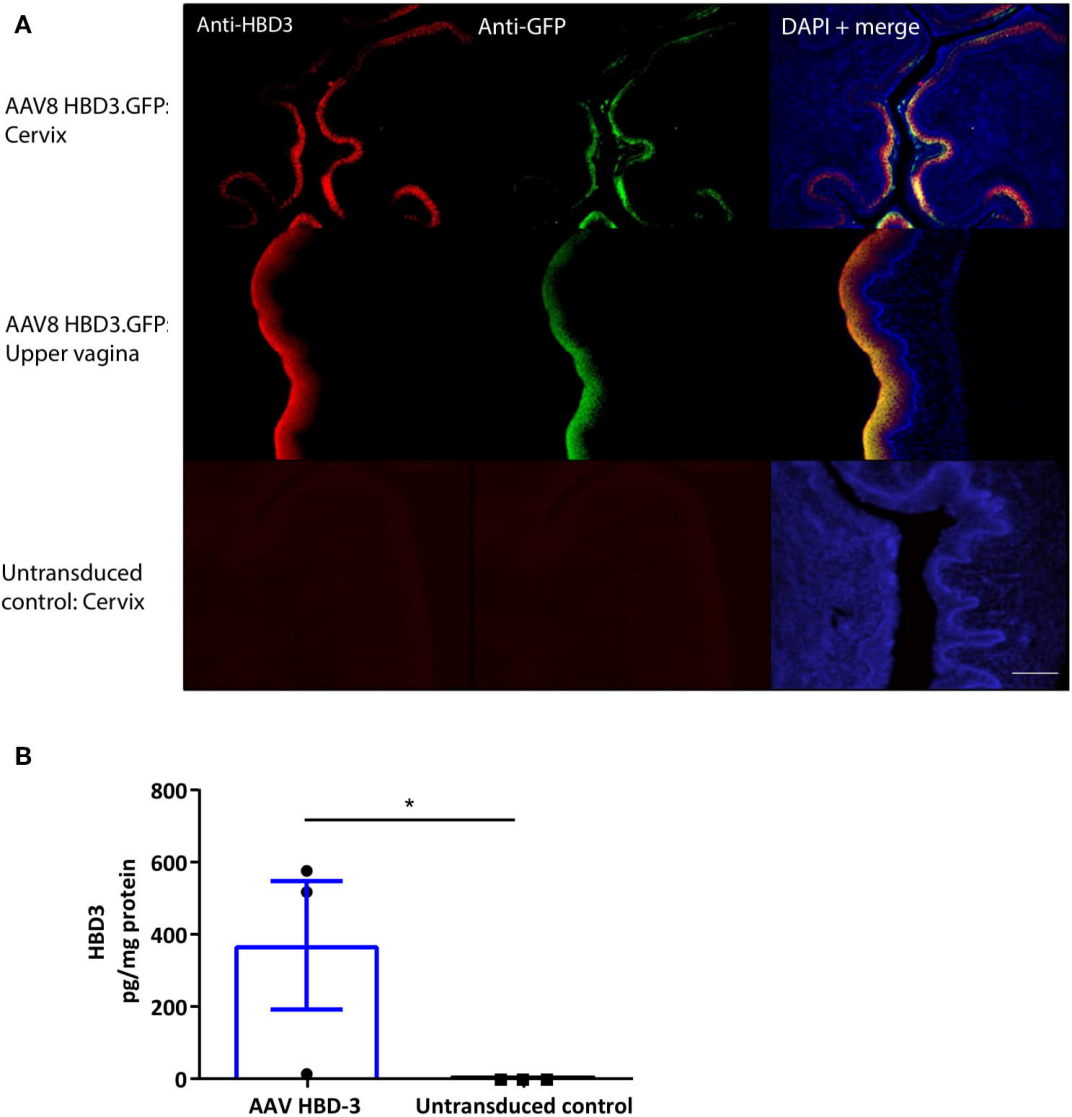
Cervical delivery of the AAV8 HBD3.GFP vector results in HBD3 peptide expression in the epithelial layers of the cervix and upper vagina and is secreted into the vaginal fluid. **(A)** Upper vaginal and cervical tissue were harvested 72 h after vector or PBS administration. HBD3 and GFP are detected in the cervix and vagina following AAV HBD3.GFP administration, *n* = 3. Scale bar 20 μm. **(B)** Vaginal lavage was collected from a different cohort of mice 72 h after administration of the vector or PBS, *n* = 3. ^*^P < 0.05, data were analyzed by an unpaired *T*-test. The cross-reactive non-specific background from the ELISA was not subtracted from our analysis.

Following intravaginal administration of AAV8 HBD3.GFP, HBD3, and GFP expression was co-localized in the cervical and upper vaginal epithelium after 72 h (Figure 3A). HBD3 was detected in the vaginal lavage 96 h after vector administration, indicating that the HBD3 peptide was appropriately synthesized and secreted into the mouse cervix and vagina (Figure 3B).

**Figure 4:**
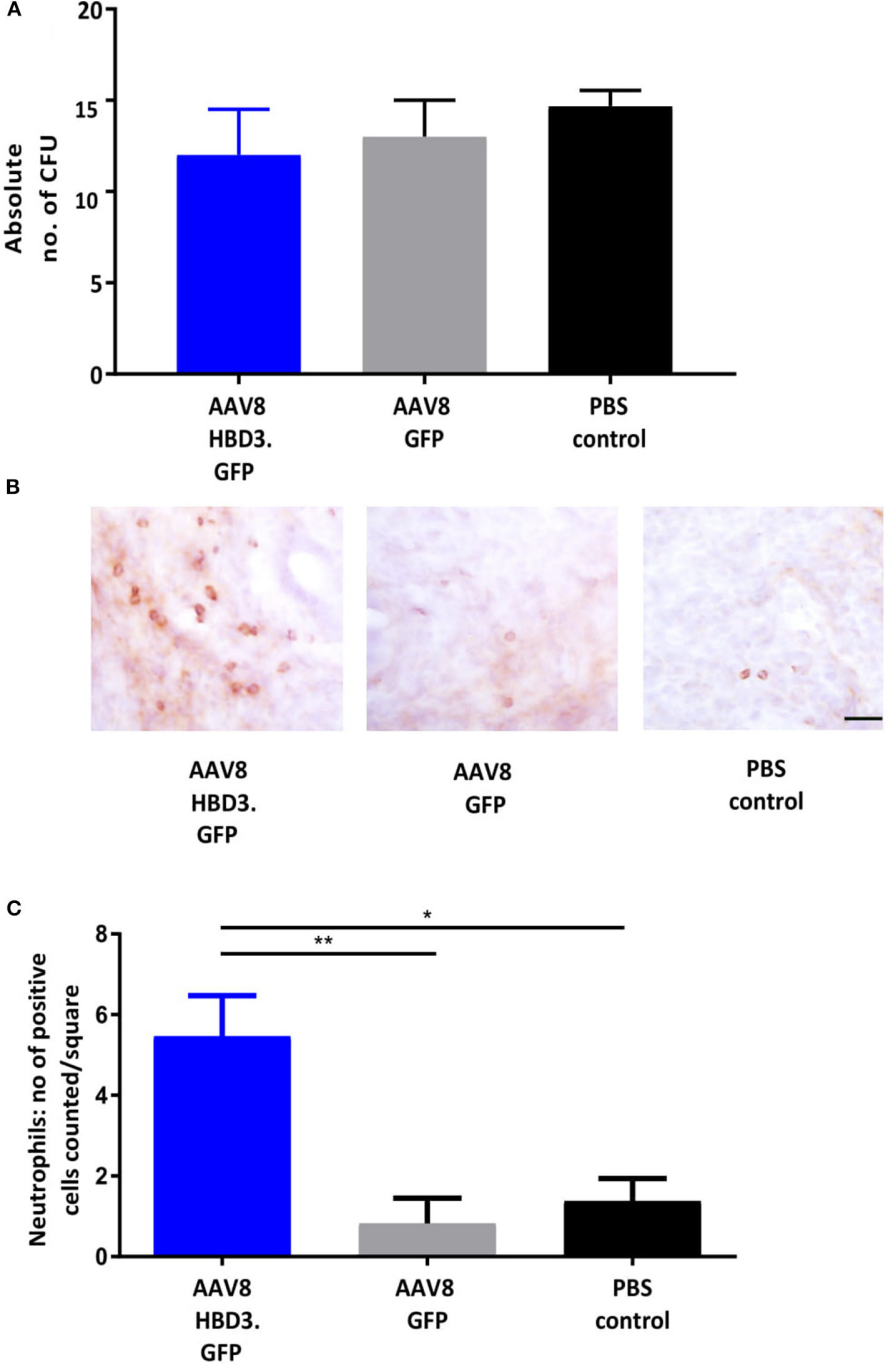
AAV8 HBD3.GFP increases neutrophil recruitment to the upper cervical epithelial layers. **(A)** *Escherichia coli* killing assays were performed on vaginal lavages from AAV8 HBD3.GFP, AAV8 GFP, and PBS treated mice. There was no difference in bacterial kill, *n* = 3. Data were analyzed with a 1-way ANOVA and *post-hoc* Bonferroni tests. **(B)** Representative images of cervical neutrophil localization using immunohistochemical staining of Ly-6g with haematoxylin counterstain. Brown coloration depicts DAB-positive cells, Scale bar 20 μm. **(C)** Neutrophil numbers in cervical epithelial cell layers were increased following AAV8 HBD-3 transduction, compared with AAV8 GFP (*P* = 0.009) and PBS (*P* = 0.02); AAV8 GFP vs. PBS (*P* = 0.99), *n* = 3. ^*^P < 0.05, ^**^P < 0.01, data were analyzed with a 1-way ANOVA and *post-hoc* Bonferroni tests.

We performed *E. coli* killing assays with these lavages. A trend for increased bactericidal activity was observed in AAV HBD3.GFP treated vs. PBS controls, but this did not reach statistical significance (Figure 4A). Immunohistochemical detection of neutrophils was performed on cervical tissue 72 h after AAV.HBD3 GFP, AAV.GFP and PBS intravaginal administration (Figure 4B). This showed a significant increase in neutrophil recruitment to the cervical epithelium layer in the AAV8 HBD3.GFP group 72 h after vector administration, compared with the AAV8 GFP and PBS only controls (*P* = *0.009* and *P* = *0.017*, respectively, Figure 4C).

Vaginal lavage samples were taken before- and at multiple time points after- vector or PBS administration to investigate the effect of HBD3 on the vaginal microbiome. Delivery of AAV8 HBD3.GFP had no effect on the alpha diversity index (Supplementary Figure 2A) or the distribution of bacterial classes compared to the AAV8 GFP control group was similar before and after vector administration (Supplementary Figure 2B).

### HBD3 Gene Delivery to Prevent Ascending Infection

**Figure 5:**
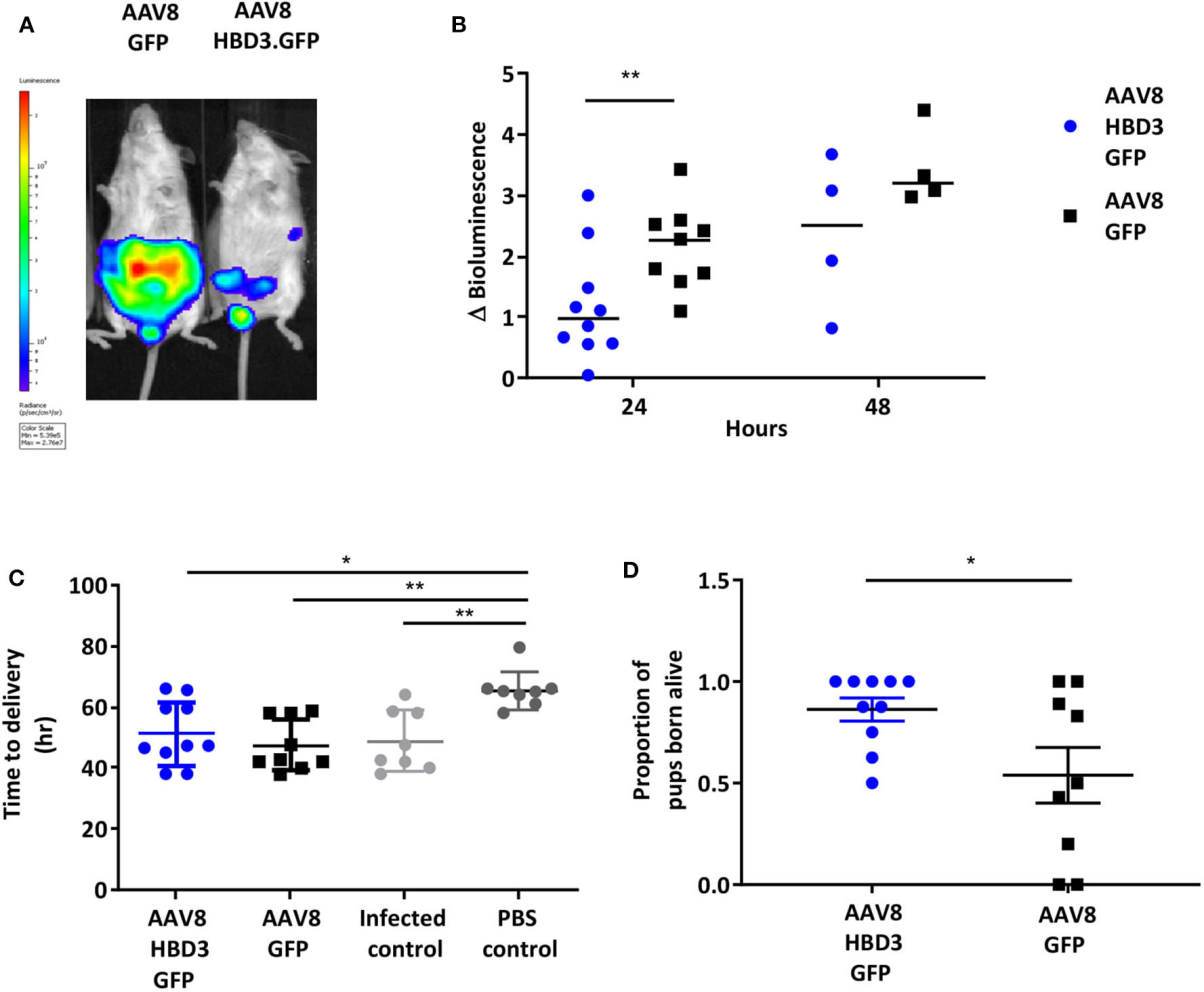
Delivery of AAV8 HBD3.GFP reduces bacterial ascent into the pregnant uterine cavity and increases the proportion of pups born alive. **(A)** An example of an AAV8 HBD3.GFP treated mouse and an AAV8 GFP treated mouse 24 h after bacterial administration. **(B)** Marked difference in uterine bacterial bioluminescence in mice treated with AAV8 HBD3.GFP, compared with AAV8 GFP, *n* = 10 at 24 h (*P* = 0.009); *n* = 4 at 48 h (*P* = 0.09); smaller sample size as postnatal dams were excluded. ^**^P < 0.01, data were log-transformed and analyzed with unpaired *t*-tests due to sample size differences at the 2 time points. **(C)** The AAV8 HBD3.GFP group delivered significantly earlier than the control mice not infected with bacteria (PBS only) and the mean time of delivery was not significantly different from that of the AAV8 GFP group or the infected only group, *n* = 8–10. ^*^P < 0.05, ^**^P < 0.01, data were log transformed and analyzed with a one-way ANOVA and *post hoc* Bonferroni tests. **(D)** There was an increase in the proportion of pups born alive in the AAV8 HBD3.GFP treated group, compared with the AAV8 GFP group, *n* = 10. ^*^P < 0.05, data were arc-sin transformed and analyzed with an unpaired test.

Next we investigated the effects of AAV8 HBD3.GFP delivery on ascending infection and preterm birth. AAV8 HBD3.GFP or AAV8 GFP control was administered to the vaginas of pregnant dams on embryonic day 13.5, followed by administration of *E. coli* K1 intravaginally on embryonic day 16.5. Representative images of an AAV8 HBD3.GFP treated and AAV8 GFP control mouse are shown in Figure 5A. AAV8 HBD3.GFP administration resulted in significantly less uterine bacterial bioluminescence, a marker of bacterial ascent, 24 h after bacterial administration (embryonic day 17.5) (*P* = *0.0015*). A similar trend at 48 h post-bacterial administration (embryonic day 18.5) was seen but did not reach statistical significance possibly due to reducing levels of HBD3 expression by this time point (*P* = *0.09*; Figure 5B).

The reduced ascent of bacteria into the uterine cavity in the AAV8 HBD3.GFP group led us to hypothesize that this group would also have reduced preterm birth rates. Delivery within 48 h (embryonic day 18.5) of intravaginal administration of *E. coli* K1 was considered preterm birth, whereas mice delivering after this point was considered term. AAV8 HBD3.GFP resulted in a small, non-significant reduction in preterm labor to 60 vs. 78% in AAV8 GFP controls (*P* = *0.37*, Table 1).

**Table 1:** Ascending infection-induced preterm birth rates: preterm birth rates after intravaginal administration of *Escherichia coli* K1 on embryonic day 16.5.

	**PTB rates**	**n**
AAV8 HBD3.GFP	60%	10
AAV8 GFP	78%	9

*Data analyzed with Fisher exact test*.

The AAV8 HBD3.GFP group delivered significantly earlier than the control mice not infected with bacteria (mean 50.7 h ± 11.4 vs. 65.5 h ± 6.3, *P* = *0.02*, Figure 5C) and the mean time of delivery was not significantly different from that of the AAV8 GFP group or the infected only group (mean 50.7 h ± 11.4 vs. 46.1 ± 8.0 vs. 48.81 ± 6.2, *P* = *0.99*, Figure 5C).

**Figure 6:**
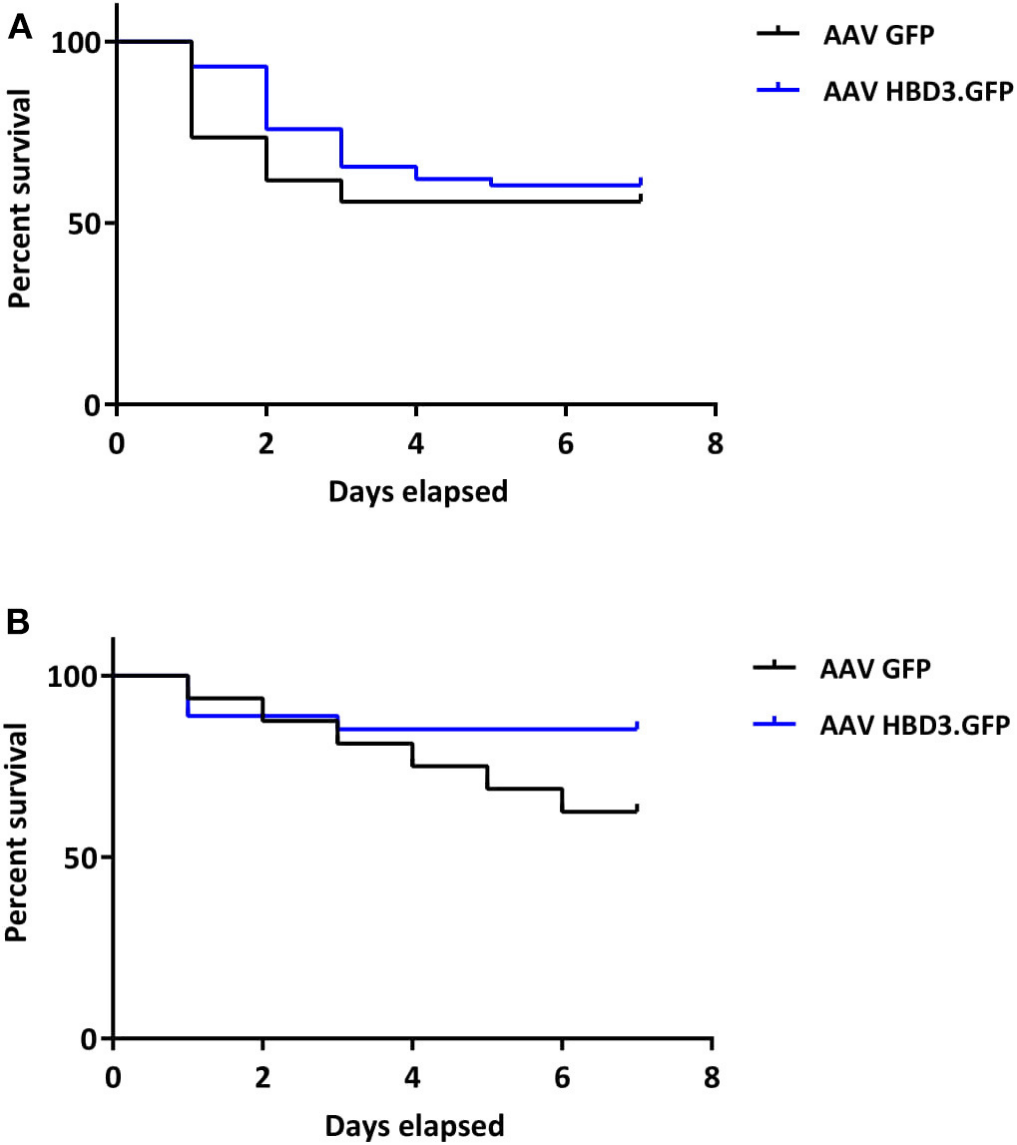
There is no difference in pup survival over the first week of life between AAV HBD3.GFP and AAV GFP pups, although there is a trend for increased survival in the term AAV HBD3.GFP pups compared with AAV GFP controls pups. **(A)** Overall pup survival over the first week of life (all pups were culled on Day 7). **(B)** Term pup survival over the first week of life (all pups were culled on Day 7). Term pups were defined as those that were delivered > 48 h after bacterial administration. *n* = 53 in the AAV HBD3.GFP group, *n* = 27 in AAV GFP group (Term pups; AAV HBD3.GFP *n* = 23, AAV GFP *n* = 10). Data analyzed by Log-rank Mantel-Cox test.

AAV HBD3.GFP treatment significantly increased the proportions of live pups, vs. AAV8 GFP controls (0.86 vs. 0.54, respectively (Figure 5D), Unpaired *t*-test; *P* = 0.028) but did not increase survival rates at 7 days (AAV8 HBD3.GFP 60.3% vs. AAV8 GFP 55.9%, *P* = 0.2; Figure 6A). However, there was a small non-significant increase in survival in those pups born at term in the AAV8 HBD3.GFP group (85.2 vs. 62.5%, respectively, *P* = 0.16, Figure 6B).

## Discussion

Despite recent advances in preterm birth research, current therapies do not appear to have an impact on preterm birth rates (38). Approximately 40% of preterm births are thought to be associated with infection, specifically ascending infection from the vagina and despite this knowledge, prophylactic antibiotics have not be shown to alter preterm birth rates (39). Here, we show that a localized cervical treatment, which delivers an endogenous human gene with antimicrobial properties, can reduce ascending infection in pregnant mice and lead to an increase in neonatal survival.

We show that gene delivery to the murine cervix is possible using common viral vectors and is augmented by the use of thermosensitive pluronic gel. In contrast to the human cervix, the entirety of the murine cervix comprises stratified squamous epithelium (40). Although little is known about the mouse cervix, we do know that one layer of the 28-layer human vaginal and ectocervical stratified squamous epithelium sheds every 4 h (41) and so pluronic gels may improve transduction efficiencies by increasing contact time of vector with epithelium. Pluronic gels have previously been used in combination with adenovirus vector to deliver VEGF to the uterine circulation as a treatment for fetal growth restriction (42). The benefits of these gels are that they are biodegradable, low in toxicity and they transfer from aqueous phase to gel phase on increasing temperature. Furthermore, there is evidence that pluronic gels help to facilitate cervico-mucus plug penetration without comprising the function of the barrier (43).

AAV8 HBD3.GFP did not significantly increase the gestational length in *E. coli* K1 infected dams. However, there was an increase in the proportion of pups born alive from dams in this group with a tendency toward increased 7 day survival rates for neonates born at term following *E. coli* K1 infection. There was no increase in preterm pup survival in the first week of life, however, suggesting that pups delivered early are more susceptible to *E. coli* K1 infection. Although *E. coli* is not classically associated with preterm birth, it has commonly been used to induce preterm birth in animal models. The pathogenic strain used in this study, *E. coli* K1 strain A192PP, like group B streptococcus, is responsible for causing neonatal meningitis in humans which is dependent on vertical transmission from the mother44. In preterm birth, the bacteria identified include a wide array notable for relatively low levels of pathogenicity such as Ureaplasma spp. and Mycoplasma spp6. Experiments performed by our group using non-pathogenic *E. coli* K12 do indeed show reduced bacterial ascent into the uterine cavity following cervical HBD-3 therapy, however, this model does not induce preterm birth in mice (unpublished data) (28).

The mechanisms by which cervical HBD3 reduces bacterial ascent into the uterine cavity is uncertain. Although HBD3 is known for its potent antimicrobial action, we were unable to measure a statistical difference in the microbicidal activity of the vaginal lavage from AAV8 HBD3.GFP vs. AAV8 GFP treated mice in the experimental time frame. Microbicidal activity has only been detected previously in the ng-μg/ml range (21), however our supplementary data shows some *E. coli* killing activity at pg/ml (shown in Supplementary Figure 3) and so it is unlikely that the pg/mg levels detected here are insufficient to lead to some direct antibacterial activity *in vivo*. It is possible, however, that the killing assays using the HBD3 peptide obtained from the lavage sample did not show any direct killing activity due to the instability of these peptides in bodily fluids due to peptidase degradation (44, 45). Interestingly, within the same time frame, the presence of HBD3 resulted in an increase in neutrophil influx into the cervical epithelium *in vivo*. Therefore, it is likely that HBD3-mediated bacterial clearance involves activation of multiple antimicrobial mechanism(s). A study looking at the benefits of exogenously delivered human cathelicidin LL-37 in a murine model of *Pseudomonas aeruginosa* lung infection found that cathelicidin LL-37 enhanced bacterial clearance *in vivo* (46). The authors similarly found no evidence of a direct microbicidal effect and it was concluded that the likely mechanism of bacterial clearance is peptide-mediated enhanced early neutrophil influx. Our observations also support a crucial role for HBD3-driven neutrophil influx, taken together, it is tempting to suggest that neutrophil mediation may present a significant armory of AMP action.

The HBD3 levels detected at a single time point in the vaginal lavage are incredibly small following freeze drying of the lavage (~200 pg/mg protein). In the literature only levels in the ng-μg/mg protein range from transfected cells *in vitro* have been detected on western blot (47, 48). This may be primarily be due to species variation and/or due to the experimental setup of our current study e.g., cervicovaginal samples were taken 96 h following gene transfer whilst bioluminescence (indicative of HBD3 gene expression) continued up to 120 h post-gene delivery (Figure 2A). Future experiments, exploring dose-response and time course of expression will be valuable for providing further insight into HBD3 production. Cervicovaginal levels of endogenous mouse defensin peptides were not assessed, however, our unpublished data suggests a trend toward reduced *Defb14* (the gene for mBD14, the mouse ortholog of HBD-3) mRNA levels in the AAV8 HBD-3 treated cervices. Further studies would need to be conducted to assess the level of mouse defensin peptides within the cervico-vaginal fluid.

Recent data has shown that human cervical mucus plugs contain at least 28 AMPs, although interestingly, the AMPs were at insufficient concentrations to have antimicrobial activity against Group *B streptococcus*, a bacteria commonly implicated in preterm birth (49). However, these AMP appear to have a role in amplifying the immune response including enhanced leucocyte activity and complement-mediated killing. In support of this, emerging evidence suggests that HBDs have a much more complex role in the immune system than being solely endogenous antimicrobials, they have previously been shown to recruit immune cells, such as neutrophils, macrophages, and dendritic cells (50, 51). In contrast to our findings here, HBD3 has previously been reported to have direct chemotactic activity on human monocytes and not neutrophils (51). The chemotactic activity on murine neutrophils seen in this study and its mechanism, direct or indirect, needs to be elucidated in future studies. Neutrophils are critical effector cells and form part of the first line of defense against microorganisms. HBD2 has been shown to attract neutrophils via the G-protein phospholipase C-dependent pathway (50). The cysteine residues of the three disulphide bridge structure of HBD3 appear to be necessary for this chemotactic role (52). In addition to its chemotactic role, HBD3 has been shown to prolong the lifespan of neutrophils at sites of infection by preventing apoptosis (53). The molecular mechanism(s) involved in HBD3-mediated neutrophil influx in our model system require further investigation.

During human pregnancy, a rise in estrogen levels leads to a stable vaginal microbiome dominated by *Lactobacillus* species (54). *Lactobacillus* are thought to inhibit pathogen growth by secreting antimicrobial bacteriocins as well as producing lactic acid which helps to maintain a low acidic pH (55). The composition of the vaginal microbiota appears to be closely associated with preterm birth outcome (13, 56). Mice treated with either AAV8 HBD3.GFP or AAV8 GFP did not show significant changes in their vaginal microbiota nor did it modify *Lactobacillus* species abundance in the vagina (data not shown). This is not surprising given the limited bactericidal activity seen in the lavage samples (likely as a result of insufficient levels of HBD3) and so it is likely the microbiota system is stable to minor fluctuations. We know that in human pregnancy, the vaginal microbiota becomes less diverse and more stable despite the hormonal and immunological changes associated with pregnancy (57). Furthermore, sampling 7 days following gene therapy may be an insufficient time course for detection of change as a previous study looking at the effects of stress show differences > 14 days later (58). In support of our findings, HBD3 does not exhibit antibacterial activity against lactic acid bacteria, including *L. rhamnosus*, suggesting that these bacteria may exhibit factors that protect them from host immune defense (59). Recent data indicates an association between an *L.iners*-dominant vaginal microbiome and an increased risk of preterm birth, whilst *L.crispatus*-dominance appears to be protective (13). *L.crispatus* promotes epithelial cell defense against *Candida albicans in vitro* by increasing HBD levels, in particular, HBD3 (60). This finding may play a role in linking the mechanism of *L.crispatus*-dominant vaginal microbiome with its protective effect on preterm birth.

HBD3 was previously considered to be pro-inflammatory as its expression increases following TLR activation or IL-1β, TNF-α and IFN-⋎ release (21, 51), however, emerging data recognizes it more as a multifunctional effector of the immune system (61). It is possible that HBD3 may induce a strong chemoattractant and inflammatory response at high concentrations during infection and injury to respond to the insult whilst at lower concentrations it has anti-inflammatory and healing properties (62). In this study, we found no evidence of increased inflammatory cytokine expression in the cervical tissue at 72 h following gene transfer of AAV.HBD3 GFP compared with AAV GFP but further time course expression analyses may show differences (Supplementary Figure 4). Further studies would also need to be conducted to assess for other surrogate markers of inflammation or cytotoxicity, such as NF-kB activation, particularly as HBD-3 has been found to be overexpressed in cases of cervical cancer (63). In the GI tract, HBD3 plays an important role in contributing to mucosal immune tolerance (64). As the vaginal microbial composition appears to be so closely related to preterm birth risk, HBD3 expression in the cervix may also improve immune tolerance to certain commensal bacteria.

The use of antimicrobial gene therapy has been explored previously, mainly as a way of treating infections with antibiotic resistant bacteria and treating certain wound infections where the skin epithelium has been severely damaged, such as burns (65). Gene delivery of HBD3 to keratinocytes using adenoviral vectors has been performed in studies investigating novel treatments for wound infections (66, 67). In an *in vivo* porcine model of infected diabetic wounds, adenoviral vectors delivering HBD3 resulted in reduced bacterial load 4 days after vector administration and an improvement in re-epithelialization of the wound (66). The wound healing role of HBD3 may also be of interest in women at high risk of preterm birth who have had previous cervical treatment for high risk precancerous cervical lesions. In particular, HBD3 is shown to promote skin re-epithelialization, granulation tissue formation and collagen I deposition. In preterm birth, premature remodeling, and shortening of the cervix involves degradation of cervical stromal collagen, particularly collagen I and III, by matrix metalloproteinases (68–70). Therefore, HBD3 gene therapy could help to inhibit this process by encouraging collagen I deposition.

Preterm birth remains a major global health problem, being responsible for greater than a million neonatal deaths per year (71). Despite the employment of current preventative strategies for preterm birth, there has been no decline in preterm birth rates highlighting the importance of further research into novel therapies (72). The use of HBD3 in augmenting cervical mucosal immunity may have a role in reducing the intra-uterine inflammation associated with ascending vaginal infection-related preterm birth and warrants further studies to explore its potential for clinical translation.

## Data Availability Statement

The datasets generated for this study are available on request to the corresponding author.

## Ethics Statement

All animal studies were conducted under UK Home Office license 70/8030 and approved by the UCL Ethical Review Committee.

## Author Contributions

NS, RK, JN, JB, DPer, and JD performed experiments. NS, SB, DA, MB-E, PT, SW, and DPee designed experiments, analyzed, and interpreted data. NS wrote the manuscript. SW, DPee, and MB-E edited the manuscript.

### Conflict of Interest

The authors declare that the research was conducted in the absence of any commercial or financial relationships that could be construed as a potential conflict of interest.

## Abbreviations

PTB: preterm birth
HBD-3: human β-defensin-3.
