# Supplementary figures and images for: Cervical Gene Delivery of the Antimicrobial Peptide, Human β-Defensin (HBD)-3, in a Mouse Model of Ascending Infection-Related Preterm Birth
Source: Front Immunol. 2020 Feb 11;11:106. doi: 10.3389/fimmu.2020.00106 (PMC7026235; doi:10.3389/fimmu.2020.00106)

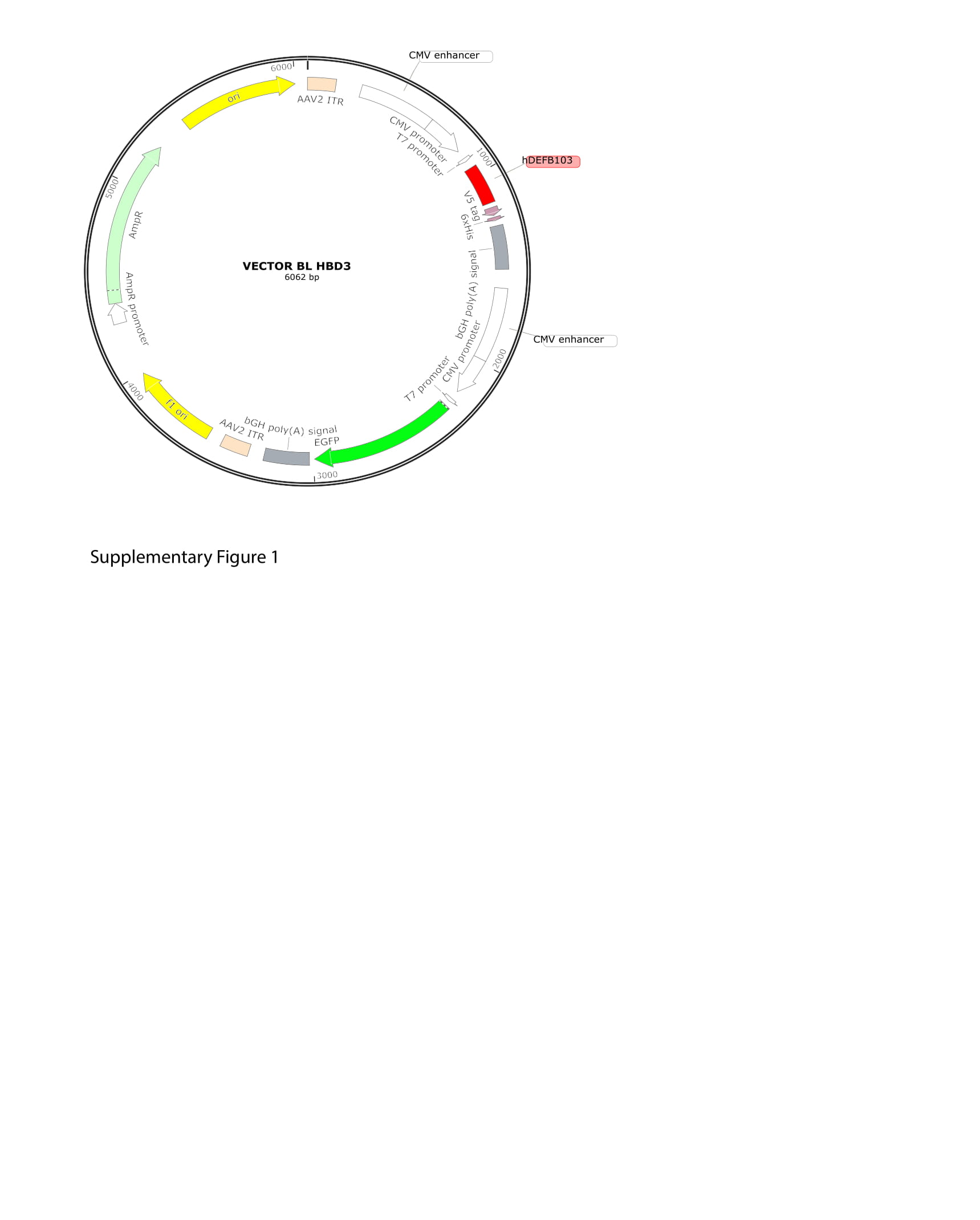

Supplement: Supplementary file 1 [file Data_Sheet_1.zip › Figure 1.JPEG]

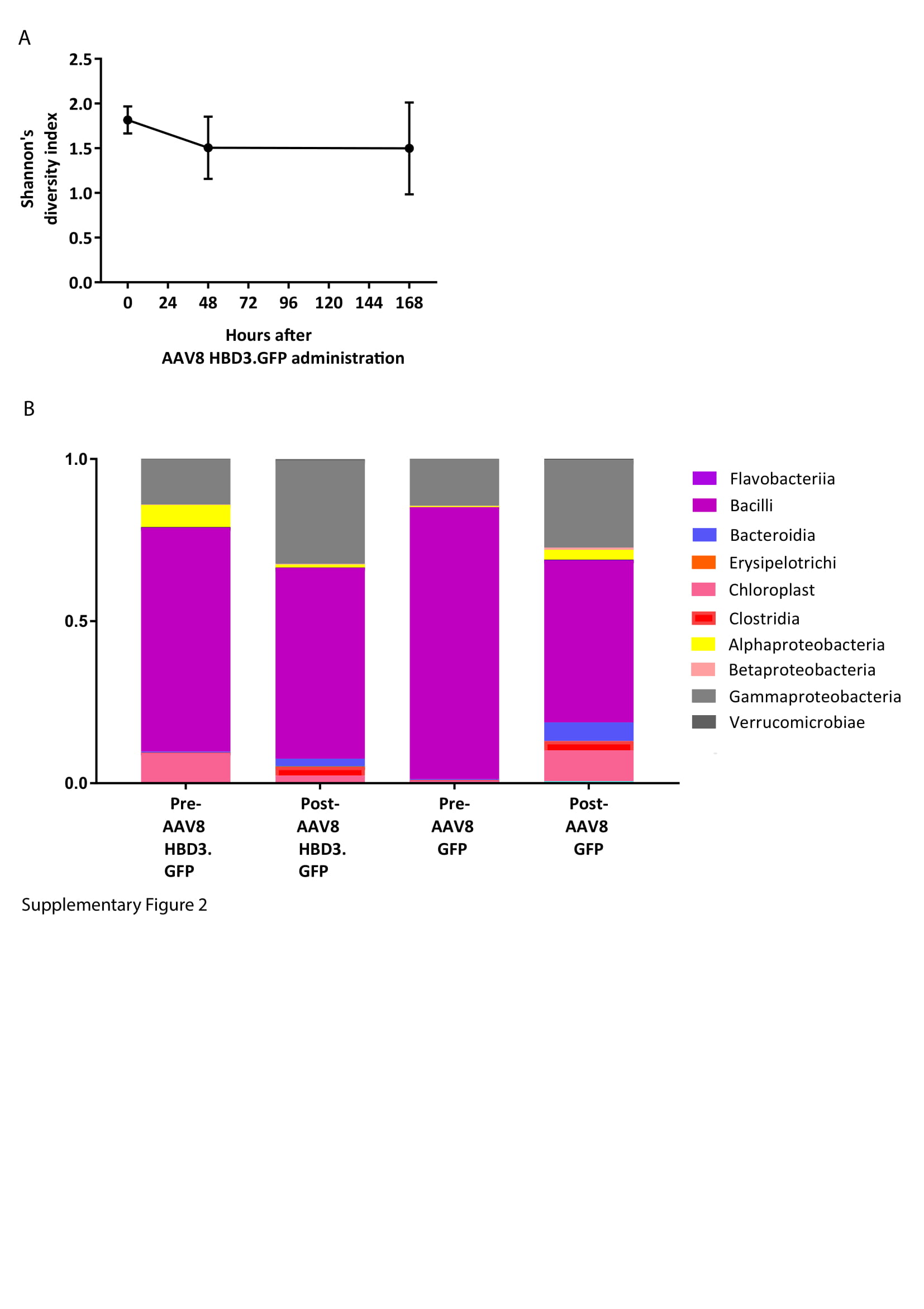

Supplement: Supplementary file 1 [file Data_Sheet_1.zip › Figure 2.JPEG]

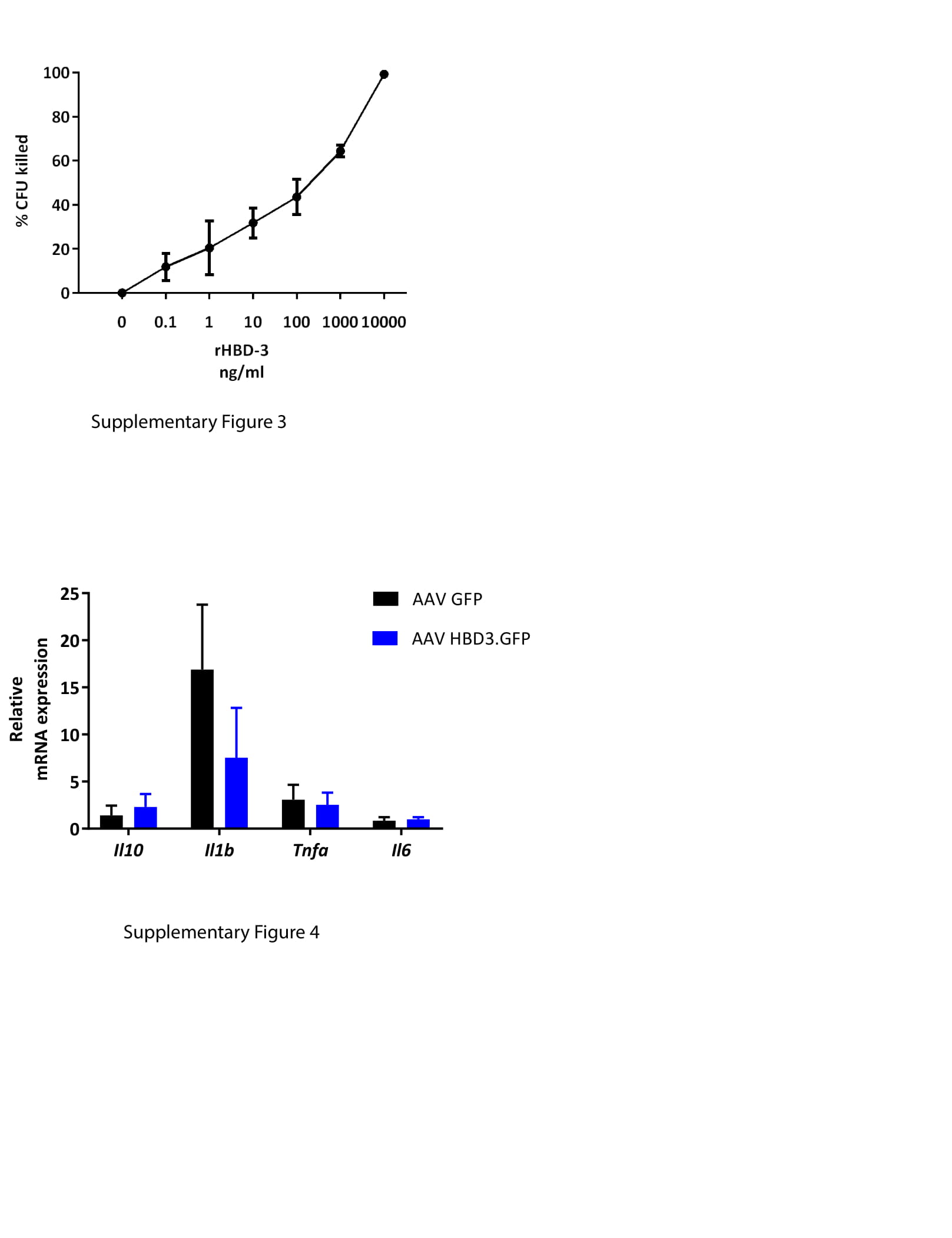

Supplement: Supplementary file 1 [file Data_Sheet_1.zip › Figures 3 and 4.JPEG]
