# Supplementary material for: Cervical Gene Delivery of the Antimicrobial Peptide, Human β-Defensin (HBD)-3, in a Mouse Model of Ascending Infection-Related Preterm Birth
Source: Front Immunol. 2020 Feb 11;11:106. doi: 10.3389/fimmu.2020.00106 (PMC7026235; doi:10.3389/fimmu.2020.00106)
Supplement: Supplementary file 2 [file Data_Sheet_2.pdf]

Supplementary Figure 1. AAV HBD3.GFP construct. An AAV8 bicistronic vector encapsidating a single-stranded DNA sequence containing the HBD3 gene (Vega Sanger HBD3 transcript VEGA68:CM000670.2) under the transcription activity of the CMV promoter, followed by the eGFP gene under the transcription activity of a further CMV promoter, and BGH polyA downstream (Vector Biolabs, Malvern, USA).

Supplementary Figure 2. AAV HBD3.GFP does not have an effect on the vaginal microbiome. (A) Alpha diversity was determined before, at 48 hours and at 168 hours after AAV8 HBD3.GFP administration, n=5. There was no difference in alpha-diversity values after AAV8 HBD3.GFP, compared with samples taken before AAV8 HBD3 administration. Data were analysed by a 1-way ANOVA with post hoc Bonferroni tests to before AAV8 HBD3.GFP samples. (B) The proportion of bacterial classes present in the vaginal microbiome was determined before and 168 hours (7 days) after AAV8 HBD3.GFP or AAV8 GFP administration, n=5.

Supplementary Figure 3. *E.coli* K1 kill increases with increased concentration of recombinant HBD-3. Total colony count on control plates with media alone was considered as 0%.

Supplementary Figure 4. AAV HBD3.GFP does not have an inflammatory cytokine effect on cervical tissue at 72 hours following administration, compared with AAV GFP. Relative mRNA expression was determined in AAV HBD3 and AAV GFP cervices. n=6; data shown as $2^{-\Delta CT}$ data and analysed by two-way ANOVA with post hoc Bonferroni tests.

Supplementary Table 1: qPCR primer sets

	Sequence
Gapdh<br>F<br>R	 5'-ACTCCACTCACGGCAAATTC-3'<br>5'-TCTCCATGGTGGTGAAGACA-3'
Il1b<br>F<br>R	 5'-CAGGCAGGCAGTATCACTCA-3'<br>5'-AGCTCATATGGGTCCGACAG-3'
Tnfa<br>F<br>R	 5'-TATGGCTCAGGGTCCAACTC-3'<br>5'-CTCCCTTTGCAGAACTCAGG-3'
Il6<br>F<br>R	 5'-AGTTGCCTTCTTGGGACTGA-3'<br>5'-TCCACGATTTCCCAGAGAAC-3'
Il10<br>F	

R	5’-<br>GGTGAGAAGCTGAAGACCCT-<br>3’<br>5’-<br>TGTCTAGGTCCTGGAGTCCA-<br>3’
